# Operationalizing Equity, Inclusion, and Access in Research Practice at a Large Academic Institution

**DOI:** 10.1007/s11606-023-08539-z

**Published:** 2024-02-01

**Authors:** Emma Britez Ferrante, Shira Blady, Dorothy Sheu, Medha Romee Maitra, Josiah Drakes, Adina Lieberman, Adam Mussell, Elizabeth F. Bair, Caleb M. Hearn, Leo Thorbecke, Jingsan Zhu, Rachel Kohn

**Affiliations:** 1 Palliative and Advanced Illness Research (PAIR) Center at the University of Pennsylvania, Philadelphia, PA USA; 2 University of Georgia, Athens, GA USA; 3 Xavier University of Louisiana, New Orleans, LA USA; 4 Center for Health Incentives and Behavioral Economics (CHIBE) at the University of Pennsylvania, Philadelphia, PA USA; 5 Department of Medical Ethics and Health Policy, Perelman School of Medicine at the University of Pennsylvania, Philadelphia, PA USA; 6 Department of Computer and Information Science, University of Pennsylvania, Philadelphia, PA USA; 7 Center for Digital Health, Penn Medicine Center for Health Care Innovation, Perelman School of Medicine at the University of Pennsylvania, Philadelphia, PA USA; 8 Department of Medicine, Perelman School of Medicine at the University of Pennsylvania, Philadelphia, PA USA; 9 Leonard Davis Institute of Health Economics, Perelman School of Medicine at the University of Pennsylvania, Philadelphia, PA USA

**Keywords:** diversity, equity, and inclusion, health equity, social justice, research priorities, practice guidelines

## Abstract

**Introduction:**

Healthcare advances are hindered by underrepresentation in prospective research; sociodemographic, data, and measurement infidelity in retrospective research; and a paucity of guidelines surrounding equitable research practices.

**Objective:**

The Joint Research Practices Working Group was created in 2021 to develop and disseminate guidelines for the conduct of inclusive and equitable research.

**Methods:**

Volunteer faculty and staff from two research centers at the University of Pennsylvania initiated a multi-pronged approach to guideline development, including literature searches, center-level feedback, and mutual learning with local experts.

**Results:**

We developed guidelines for (1) participant payment and incentives; (2) language interpretation and translation; (3) plain language in research communications; (4) readability of study materials; and (5) inclusive language for scientific communications. Key recommendations include (1) offer cash payments and multiple payment options to participants when required actions are completed; (2) identify top languages of your target population, map points of contact, and determine available interpretation and translation resources; (3) assess reading levels of materials and simplify language, targeting 6th- to 8th-grade reading levels; (4) improve readability through text formatting and style, symbols, and visuals; and (5) use specific, humanizing terms as adjectives rather than nouns.

**Conclusions:**

Diversity, inclusion, and access are critical values for research conduct that promotes justice and equity. These values can be operationalized through organizational commitment that combines bottom-up and top-down approaches and through partnerships across organizations that promote mutual learning and synergy. While our guidelines represent best practices at one time, we recognize that practices evolve and need to be evaluated continuously for accuracy and relevance. Our intention is to bring awareness to these critical topics and form a foundation for important conversations surrounding equitable and inclusive research practices.

**Supplementary Information:**

The online version contains supplementary material available at 10.1007/s11606-023-08539-z.

## INTRODUCTION

Diversity, inclusion, and access are critical values for research conduct that promotes justice and health equity. Healthcare equity research frequently focuses on strategies to address the impacts of structural racism on health outcomes, e.g., antiracist approaches to clinical practice.^1–3^ Diversity and inclusion are often evaluated relative to healthcare workforces or healthcare organizational culture.^4–6^ Literature on equitable or inclusive research practices themselves are sparse and focus on specific domains such as recruiting research participants who are representative of the patient population.^7^ Few resources discuss equitable and inclusive approaches broadly, despite the potential impact such work could have on all research disciplines.

For example, prospective studies are hindered by underrepresentation of women, racial and ethnic minority populations, and participants with limited English proficiency.^8–10^ Retrospective studies lack standard definitions for sociodemographic characteristics, have poor data and measurement fidelity, inappropriately treat sociodemographic characteristics as variables, unjustly center studies on white participants, and are unable to measure effects of structural racism on individual health.^11–14^

These challenges are often unique to the context in which research is conducted. Therefore, initiatives to address them may be localized and occur in parallel to similar work in other departments or institutions. Without existing published evidence, organizations that wish to address these challenges must re-initiate similar processes in their own investigations.

Therefore, the Joint Research Practices Working Group (JRP) was created in 2021 with the objective to develop and disseminate best practices and guidelines for the conduct of inclusive, equitable, and accessible research for our research centers as well as the larger healthcare research community. These guidelines are intended to address challenges faced by study teams and barriers for participants that arise throughout the arc of the research process—from recruitment and retention in clinical trials to data analysis and dissemination. While our work is tailored to our specific context, our goal in disseminating our processes and recommendations is to promote conversation and encourage cross-organizational collaboration.

## METHODS

### Setting

The Palliative and Advanced Illness Research Center (PAIR) at the University of Pennsylvania (Penn) generates evidence to advance healthcare policies and practices to improve the lives of people affected by serious illness and remove barriers to health equity that these patients commonly face. The Center for Health Incentives and Behavioral Economics (CHIBE) at Penn is a leading scientific organization using behavioral economics to improve health. Both centers include faculty and staff engaged in NIH-, PCORI-, and foundation-funded research. Given the importance of equity, inclusion, and access in PAIR’s and CHIBE’s research domains, center leadership chartered the JRP to research and consolidate best practices in these domains and disseminate the findings.

### Participants

A Steering Committee consisting of faculty and staff directors of PAIR and CHIBE chartered the JRP. The Steering Committee selected two Co-Leads (a PAIR faculty member and CHIBE senior staff member) based on written statements of interest and offered them small stipends for one year. At inception, the JRP additionally comprised multiple staff volunteers from PAIR and CHIBE, including three clinical research coordinators, three project managers, one research analyst, and a medical librarian with health equity expertise. Membership waxed and waned with staff arrivals and departures as well as novel interest in the group’s work. One year after JRP inception, a senior-level staff member was hired to lead equity initiatives across PAIR, with part of her center-supported effort dedicated to her role as the JRP Operations Lead. Over the summers, the JRP also included student researchers through the Summer Undergraduate Mentored Research (SUMR) Program, an internship that engages talented undergraduate students from underrepresented backgrounds in health services and policy research at Penn, with the goal of creating a clinical research pipeline.^15^

### Approach

The JRP initiated a multi-pronged approach to guideline development. Led by the medical librarian, we first performed literature searches on potential topics raised by the Steering Committee. We then presented our findings and elicited feedback from PAIR and CHIBE leadership, faculty, and staff at works-in-progress meetings, which helped to narrow our scope of work and identify priority topics. Concurrently, we invited local experts to our bi-weekly JRP meetings to foster mutual learning and partnerships; these experts specialized in inclusive recruitment practices, community-based participatory research, communications, research ethics, and biostatistical health equity. In this process, we formed subgroups to investigate each priority topic and conducted interviews, focus groups, and/or engaged additional local experts (eFigure 1, eTable 1). 

## RESULTS

Based on the literature, feedback, and mutual learning, we developed guidelines for (1) participant payment and incentives; (2) language interpretation and translation; (3) plain language in research communications; (4) readability of study materials; and (5) inclusive language for scientific communications (https://pair.upenn.edu/programs/jrp, https://chibe.upenn.edu/research/joint-research-practices/). These guidelines are discussed and implemented at JRP-run workshops each fall. To supplement the written guidelines, we developed a consultative service called “Practicing Equitable Research and Knowledge Sharing (PERKS)”. PERKS assist colleagues in real-time operationalization of equitable and inclusive research practices throughout the arc of the research process. Questions and recurrent themes from PERKS continue to inform our guideline development.

### Guidelines

#### Participant Payment and Incentives

Designing and utilizing payments for research participants require an understanding of different payment types, ethics surrounding payment practices, and implications for equity and access. Payment types include expense reimbursement, compensation for time, effort, and burden, and incentives to encourage certain behaviors. Ethical considerations include coercion, undue inducement (i.e., diminishing peoples’ sensitivity to research risks), unjust inducement (i.e., preferentially increasing the burden among underserved individuals), and separating research participation and payment from clinical care. Ensuring equity and access entails providing equal payment regardless of an individual’s socioeconomic status (SES) or income and offering sufficient payment to help individuals overcome barriers to research participation.

Common payment methods include cash, checks, debit cards, prepaid cards (e.g., gift cards), and account-to-account money transfers. However, study participants of lower SES are often unbanked or underbanked and limited in payment methods available to them. Non-cash options create barriers for those who do not have a secure location to receive mail, lack access to a bank, or are experiencing housing instability.

**Figure 1:**
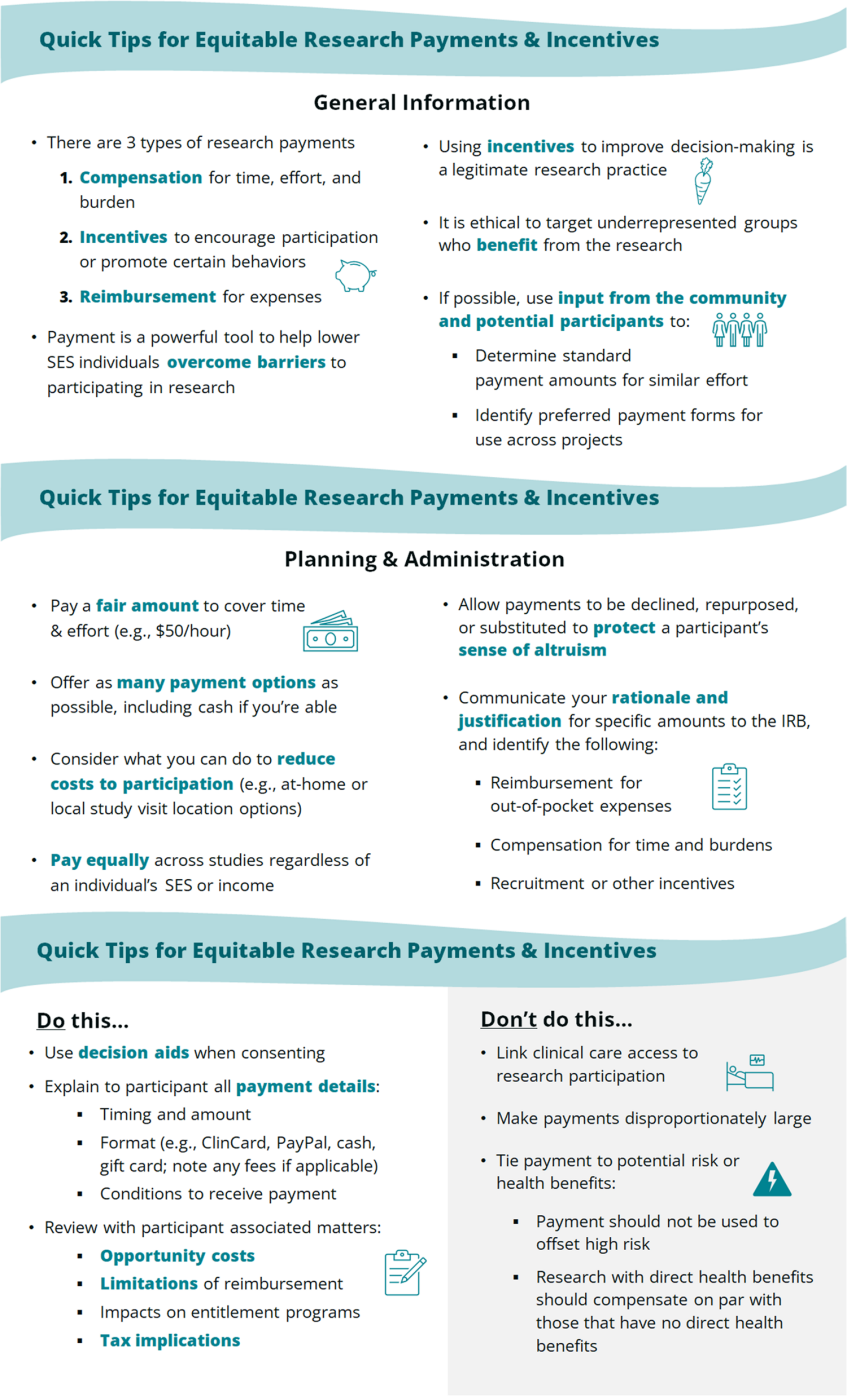
Participant payment and incentives guide.

First, we recommend that multiple payment methods (including cash) be made available to participants. Second, study teams should investigate costs and fees related to their payment methods and communicate this information to participants. Third, payments should be made as soon as the required action is completed by participants. Fourth, payment restrictions or delays should be clearly communicated to participants. Fifth, all payment should not be withheld until study completion; however, the last research visit may be paid at a higher rate to encourage study completion. Finally, researchers should proactively develop strategies to reduce participant costs, including identifying accessible study locations to reduce travel time and associated costs, and providing food, transportation, and on-site childcare or eldercare for participants (Fig. 1).^16–41^

#### Language Interpretation and Translation

In the USA, 8.4% of people speak English “less than very well,” and 22.0% of people speak a language other than English at home.^42^ Limited English proficiency (LEP) individuals are people who do not speak English as their primary language and have a limited ability to read, speak, or understand English. Most federally funded clinical trials do not provide language access (4.7% specified accommodation of other languages; 28.9% required English language proficiency), despite federal laws prohibiting discrimination and federal requirements to implement systems to provide services to LEP individuals.^10^ Excluding LEP individuals from research altogether limits the generalizability of study findings and perpetuates existing healthcare inequities; however, including LEP individuals without ensuring language access raises concerns about the soundness of informed consent, decreases participant adherence to study protocols, and is not participant-centered.

To ensure language access in your research context, identify your language needs and available services. First, identify the top languages of your target population (e.g., 2015 Language Map App: https://www.lep.gov/maps). Next, map your points of contact with participants (e.g., recruitment message, consent form, frequently asked questions). Then, determine the existing resources you can access, including interpretation and translation services. Note that *interpretation* converts spoken messages from one language into a second language; *translation* converts written messages from one language to another.

**Figure 2:**
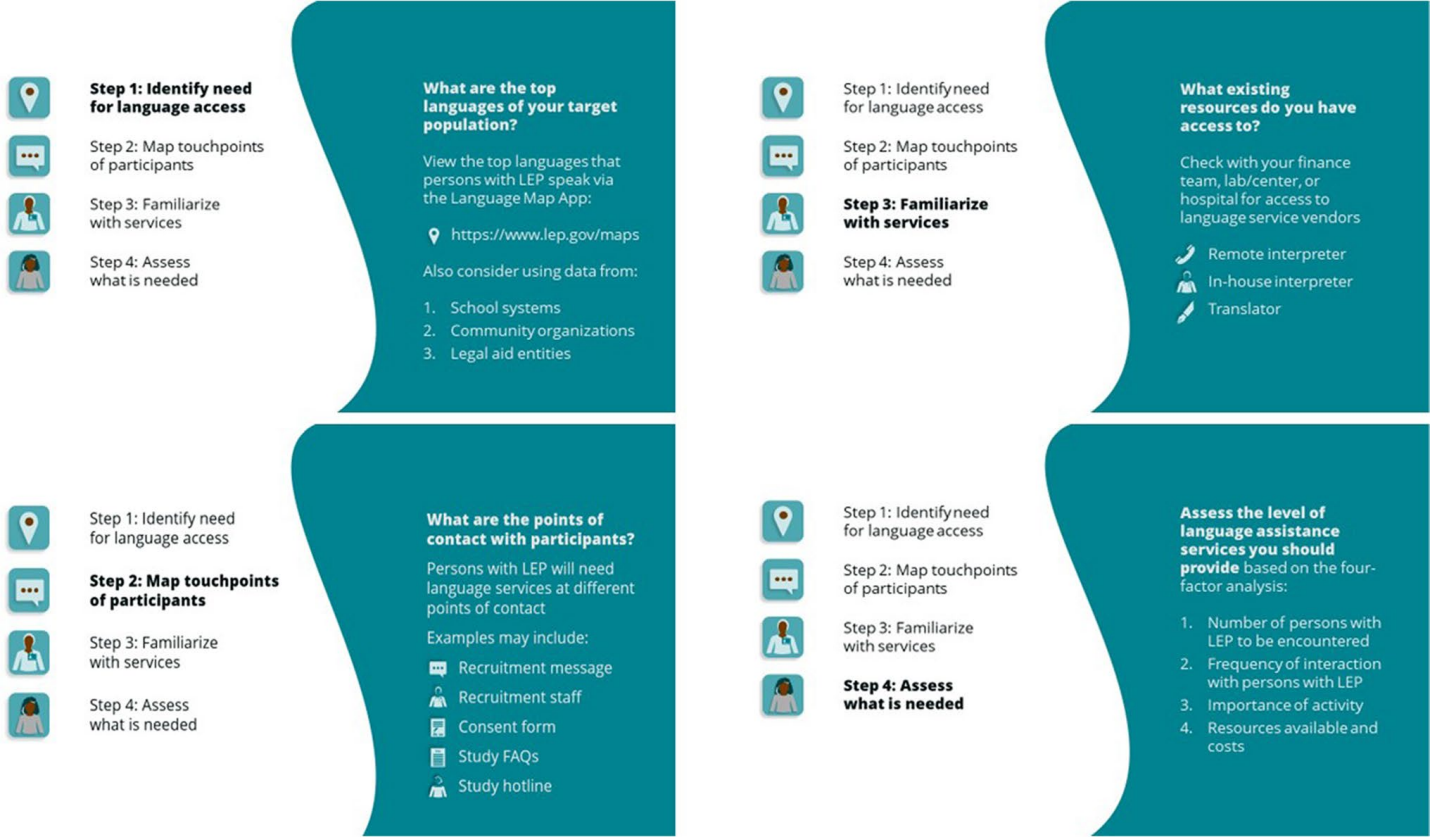
Language interpretation and translation guide.

Interpreters and translators may be remote or in-person. Remote interpreters are tested for competency, trained in healthcare terminology, and provide rapid access, but cannot always capture visual cues and gestures. In-person interpreters can build rapport and trust with participants and researchers, but have limited availability. Bilingual staff are not recommended as interpreters or translators, as they are typically untrained in interpretation and healthcare terminology and may experience conflicts of duties. Ad hoc interpreters (e.g., participants’ children) are not recommended as they too are typically untrained, make errors, change family dynamics, and interfere with confidentiality. Finally, the level of language services you provide should be based on the number of persons with LEP, frequency of interactions, research activity importance, resources available, and costs (Fig. 2).^10, 42–60^

#### Plain Language in Research Communications

Plain language makes it easier for people to find what they need, understand what they find, and use what they find to meet their needs. This is critical in research as up to 90% of US adults have limited health literacy,^61^ and approximately 30 million people in the USA have below-basic literacy,^62^ which can hamper the understanding of research.

**Figure 3:**
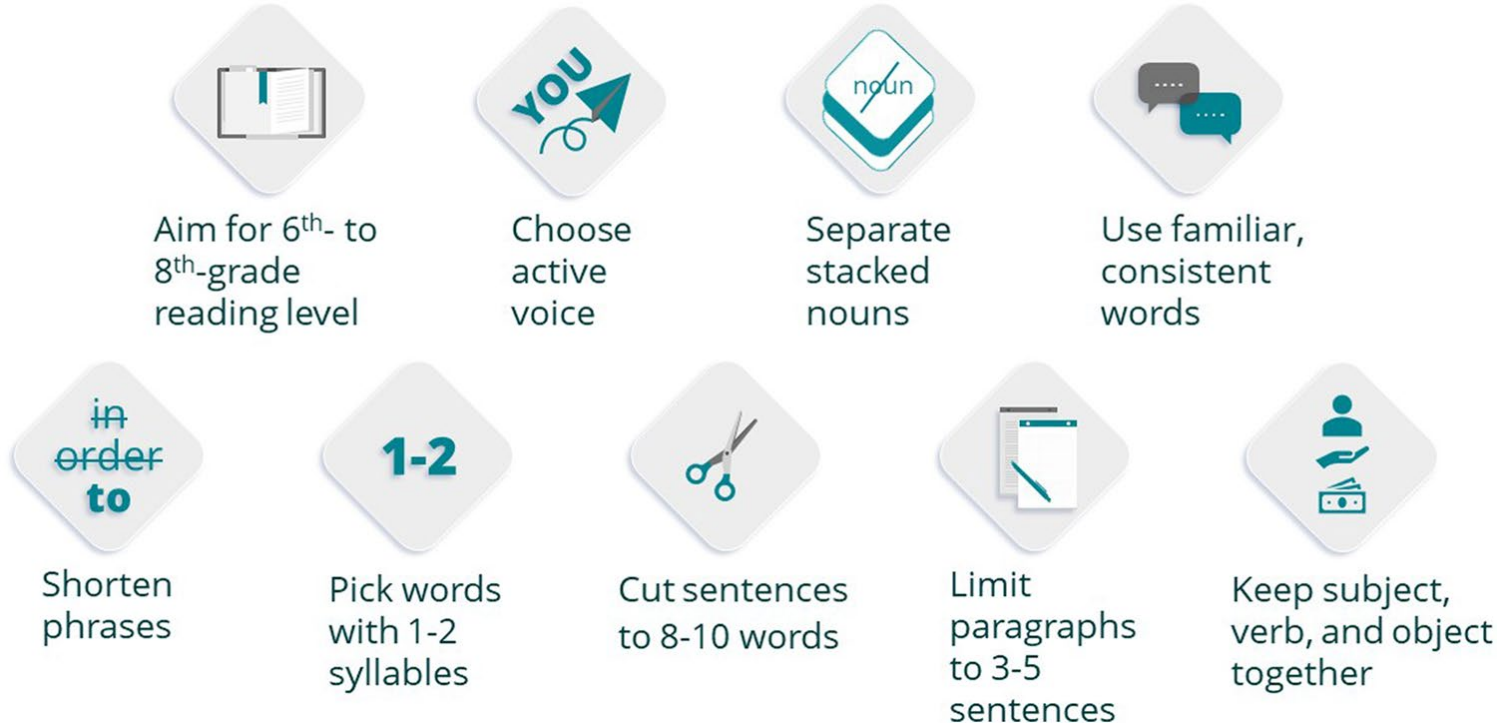
Plain language in research communications guide.

The first step in implementing plain language is to identify your audience, what you need them to do (e.g., answer a survey), and provide the information that they want to know (e.g., the time it takes to complete). The second step is to choose your words carefully. Use the following guidelines: (a) pick 1- to 2-syllable words; (b) separate stacked nouns (e.g., separated noun strings: “we are recruiting low-income patients; you need to live in Philadelphia,” rather than noun strings: “we are recruiting Philadelphia-based, low-income patients”); and (c) use familiar, consistent words. Third, design your sentences and paragraphs to (a) use active voice; (b) shorten phrases; (c) keep the subject, verb, and object close together; (d) limit sentences to 8–10 words; and (e) limit paragraphs to 3–5 sentences. Fourth, assess the reading level of your materials; a 6th- to 8th-grade reading level is appropriate for general patient populations. Various calculators are available to assess plain language (e.g., Readability Calculator: https://www.wordcalc.com/readability/) (Fig. 3).^61–69^

#### Readability of Study Materials

**Figure 4:**
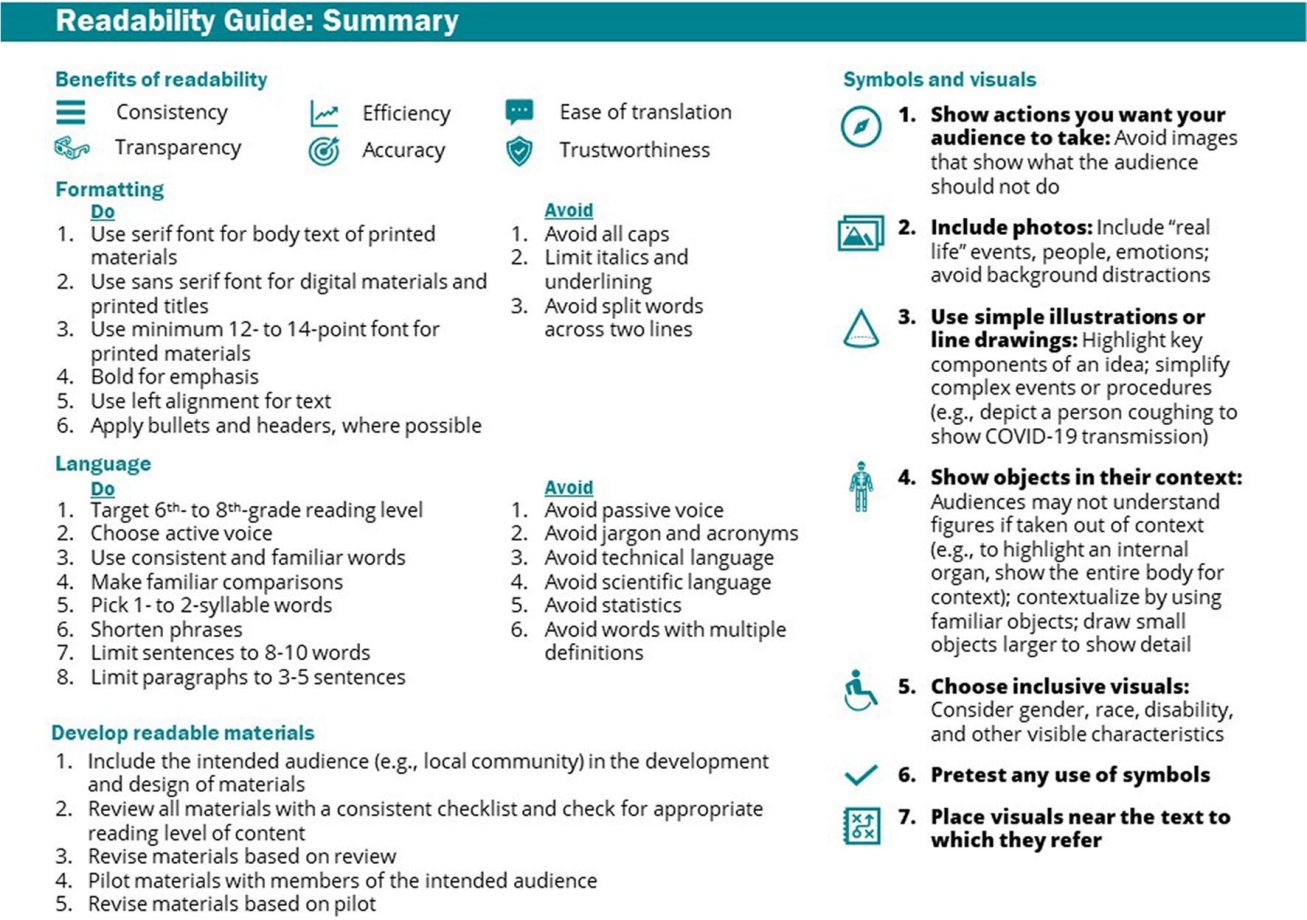
Readability of study materials guide.

Improving readability through text formatting and style, symbols, and visuals makes study materials more accessible. To format text: (1) use serif font (e.g., Times) for printed materials and sans serif font (e.g., Arial) for digital; (2) ensure minimum 12- to 14-point font size; (3) use left alignment; and (4) strategically bold text, apply bullets, and use headers. When using symbols and visuals: (1) show actions you want your participants to take rather than what they should not do; (2) include photos while avoiding background distractions; (3) use simple illustrations to highlight key components and simplify complex events; (4) show objects in their context; (5) choose inclusive visuals, considering gender, race, disability, and other visible characteristics; (6) pretest any use of symbols; and (7) place visuals near the text to which they refer (Fig. 4).^61–69^

#### Inclusive Language for Scientific Communications

**Figure 5:**
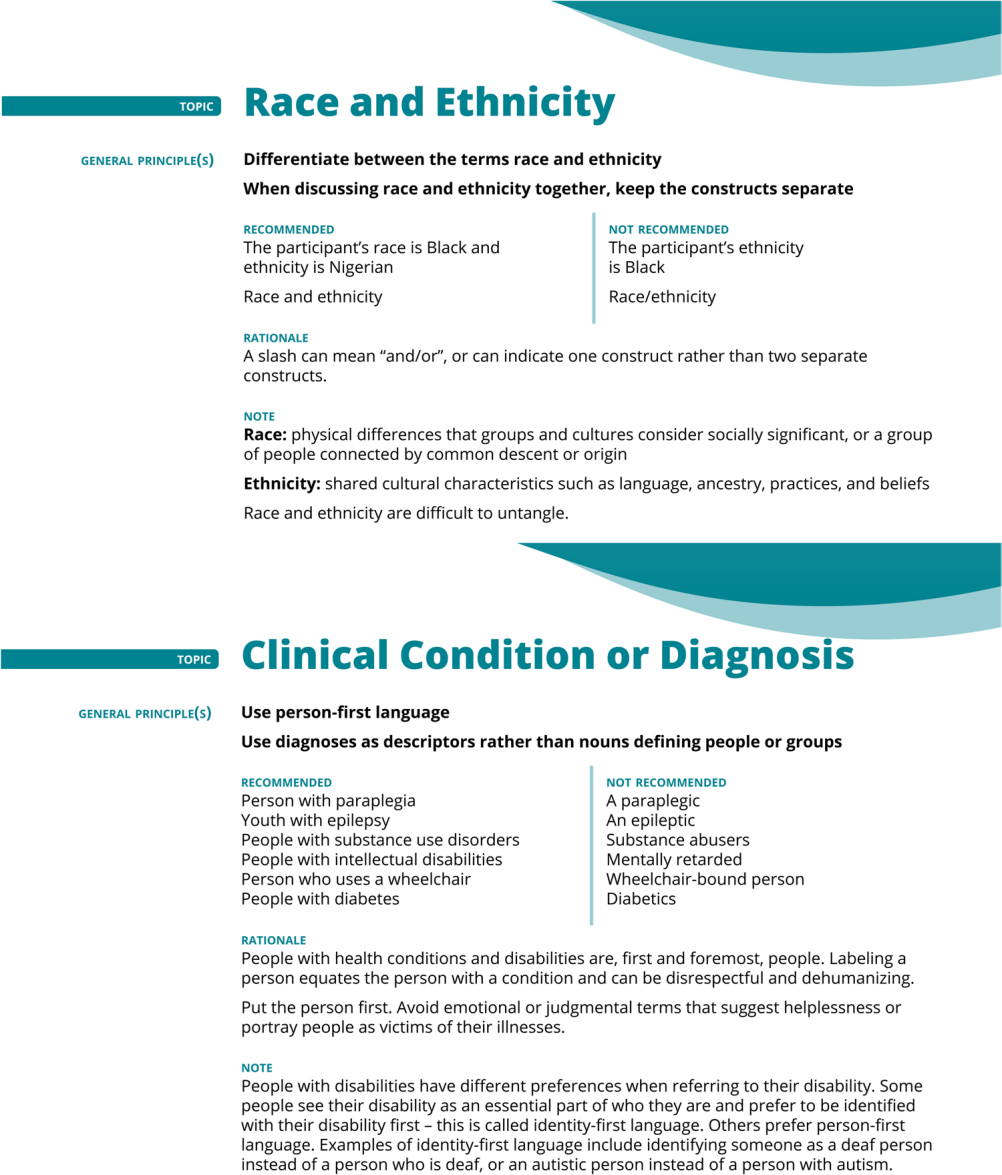
Inclusive language for scientific communications guide.

The inclusive language guide focuses on race and ethnicity, immigration status, SES, age, gender and sex, sexual orientation, and clinical conditions, primarily using guidance from the American Medical Association and Association of American Medical Colleges, American Psychological Association, GLAAD, and Americans with Disabilities Act National Network.^70–79^ Within the topic of race and ethnicity, guiding principles include (1) differentiate between the terms *race* (i.e., physical differences that groups and cultures consider socially significant) and *ethnicity* (i.e., shared cultural characteristics); (2) use words with neutral or positive connotations (e.g., “multiracial” rather than *“*mixed race”); (3) use race and ethnicity as adjectives rather than nouns (e.g., “Black patients” rather than “Blacks”); (4) spell out specific terms rather than using abbreviations; (5) avoid using the term *Caucasian* (i.e., describes people of Eurasian descent) to mean *white*; (6) differentiate between African American and Black (these terms are not interchangeable; if someone is not of American nationality, the term African American may not apply); (7) specify indigenous group or nation of origin, when applicable; (8) use the term preferred by individuals of Latin American or Hispanic descent; and (8) use terms that do not imply a white-centric perspective. For immigration status, SES, and age, the general guiding principle is to use specific humanizing terms as descriptors rather than nouns (e.g., “undocumented” rather than “illegal,” “resource-limited” rather than “the poor,” “older persons with dementia due to Alzheimer’s disease” rather than “elderly with dementia”). For gender, sex, and sexual orientation, guiding principles include (1) differentiate between gender identity and sex assigned at birth, using terms that do not portray gender identity as a choice; (2) use gender- or sex-neutral terms unless specificity is necessary (e.g., “humankind” rather than “mankind”); (3) use humanizing language when describing transgender people, in the present tense, and as an adjective (e.g., “transgender person” rather than “female-to-male transgendered person”); (4) use the term *transition* to refer to “the process a person undertakes to bring their gender expression and/or their body into alignment with their gender identity” (e.g., “she transitioned” rather than “sex change”).^70^ For clinical conditions, use person-first, humanistic language (e.g., “people with substance use disorders” rather than “substance abusers”) (Fig. 5).^70–79^

### Conference Workshops

Each fall, PAIR and CHIBE host an annual conference for faculty, trainees, and staff to share research and network across disciplines. At this conference, the JRP runs a workshop to foster discussion of timely, poignant topics regarding research diversity, equity, inclusion, and access.

In our 2022 workshop, we showed a video about Penn dermatologist Albert Kligman’s unethical research practices at the Holmesburg Prison from 1951 to 1974,^80^ and discussed an op-ed written by Adrianne D. Jones-Alston; Ms. Jones-Alston is the daughter of Leodus Jones, a Black man who was harmed by Kligman’s experiments.^81^ In small groups, participants identified the potential positive and negative impacts of research, ways to redress harm done by researchers, gaps in our knowledge and ability to identify racism and inequity, and tangible steps for preventing racism and inequity in our work (eTable 2). In the second half of the session, participants selected a themed breakout session to engage in discussions, similar to those that JRP members had cultivated over the previous year (eTable 3).

In our 2023 workshop, we re-oriented the group to the JRP’s mission and shared the guidelines developed since the prior conference. Participants selected a breakout session on one of the following: (1) study design and dissemination, (2) community engagement and stakeholder partnerships, or (3) research operations and logistics. In this breakout session, participants developed a personal commitment to incorporate a new equitable practice into their research (eTables 4 and 5).

### PERKS Consultations

Requests for PERKS consultations are fielded by the JRP Operations Lead, who then identifies JRP members and external experts to participate in the consultations. Since inception, we have conducted 32 PERKS consultations. For example, we advised critical care research teams on best practices related to interpretation and translation and brainstormed potential challenges LEP patients may face when approached to participate in prospective research. Additionally, we met with the senior operation specialists of an NIH-funded clinical trial to reduce cardiovascular disease risk and discussed the ethical deployment of higher financial incentives to community-based participants. Also, we advised a research coordinator on inclusive ways of asking demographics questions during phone-based surveys. Further, we provided guidance to a health services research assistant professor on developing a comprehensive dissemination plan for a publication on the importance of diverse clinical trial participation. Researchers can learn about PERKS through the center websites, JRP presentations, and professional networks.

Practicing research equitably is challenging. When faced with a challenge, choosing the “easy” route often means compromising equity, inclusion, and/or access. The objective of PERKS consultations is to help research teams in real-time to overcome barriers and implement strategies that keep equity at the forefront. We anticipate mounting interest in this type of support as funding and oversight agencies require researchers to demonstrate how they address equity in their studies.

## DISCUSSION

Through the JRP’s multi-year investigation, we have found a multitude of manuscripts and websites dedicated to specific topics in health equity, diversity, and inclusion in clinical practice and in the workplace, and institutional antiracism initiatives. However, there is a paucity of literature describing overall best practices for conducting health services research that is equitable, inclusive, and accessible. While the federal government, regulatory agencies, bioethicists, and social scientists have written on these topics for decades, clinical research has lagged behind. This is likely multifactorial, due to a scarcity of specific guidelines, the diffuse nature of the existing data, parallel local initiatives, a lack of knowledge and institutional research infrastructure, and insufficient grant budgeting for research practices that center equity, inclusion, and access.

As such, our guidelines are intended to be easily accessible and practical references. Coupled with PERKS consultations and recurring workshops, these guidelines facilitate conversations and the implementation of equitable practices throughout the arc of the research process. JRP members continue to engage in mutual learning and work to improve the resources available, not only to PAIR and CHIBE, but also to the larger healthcare research community.

Notably, our guidelines are informed by research completed in 2022-2023. However, we acknowledge that best practices in equity, inclusion, and access continue to evolve. This poses an ongoing challenge to identify the cadence at which information should be updated or withdrawn, and the process for doing so to ensure content remains accurate and relevant. Our intent is to bring awareness to these topics and initiate conversations surrounding equitable research practices. We affirm that how we approach research—from idea inception to publication and dissemination—matters in furthering equity, inclusion, and access.

### Limitations

First, the JRP’s productivity was limited by a primarily volunteer staff workforce. While, in theory, staff supervisors supported their supervisees volunteering for the JRP, in practice the established workweek led to JRP assignments being performed after hours or parsed to fit into available gaps in the workday. Productivity was improved by hiring the senior-level staff member as the JRP Operations Lead. Protected workweek time and dedicated finances for JRP members would likely enhance the quality, satisfaction, and sustainability of future initiatives. Second, key stakeholders, including community members, did not contribute to the development of our initial guidelines. In addition to investigating ways to elicit feedback from research participants, both PAIR and CHIBE are evaluating opportunities to engage stakeholder advisory committees. When center-affiliated partners are identified, we anticipate collaborating with them to iterate on our current guidelines and inform future content. Third, the work of promoting equity, inclusion, and access frequently falls to people of color and their allies. Often those who would benefit the most from engagement in this work are those who do not opt to participate. Fourth, national-, regional-, university-, health system-, hospital-, department-, and center-level efforts are underway to promote health equity. However, these efforts frequently occur in parallel, without resources or mechanisms to identify other organizations performing similar work. Through continued engagement, mutual learning, and knowledge-sharing with colleagues, we hope to contribute to collaboration and build synergistic efforts. Finally, this work requires cultural change and top-down infrastructure investment, including dedicated funds to support equitable research practices. Financial commitment from funding agencies at the national level and buy-in from leadership and faculty at the local level are essential for this work to become part of the fabric of our organizations.

### Future Directions

The JRP is currently developing a community engagement case study and a participant-centeredness guide. We are also developing a survey to understand research participants’ perspectives on timing of research payments, logistical barriers to receiving payments, preferred methods for payments, and how incentives influence study participation. Our goal is to embed this survey within established PAIR and CHIBE prospective studies.

We are working with other research centers, divisions, and departments to disseminate our guidelines, promote PERKS consultations, and recruit new members to join the JRP. We are partnering with the University of Pennsylvania Institutional Review Board and Office of Clinical Research to promote equitable participant payment options. We are additionally partnering with the Department of Medicine to provide interpretation services for our research studies.

Future work is needed to (1) prepare research staff to systematically and intuitively pursue equitable, inclusive, and accessible research from the outset; (2) implement equitable participant payment options; (3) provide additional resources to support participation in research (e.g., food, transportation, childcare or eldercare); (4) consistently provide interpretation and translation services across all studies; (5) meaningfully engage with community members throughout the research process; and (6) collaborate with other organizations performing similar work.

## CONCLUSIONS

Diversity, inclusion, and access are critical values for the conduct of research that promotes justice and health equity. These values can be operationalized in research through organizational commitment that combines bottom-up and top-down approaches, and through partnerships across organizations that promote mutual learning and synergy across efforts. While our guidelines represent best practices in one moment of time, we recognize that practices evolve and need to be evaluated continuously for accuracy and relevance. Our intention is to bring awareness to these critical topics and form a foundation for important conversations surrounding equitable and inclusive research practices.

### Supplementary Information

Below is the link to the electronic supplementary material.

Supplementary file1 (DOCX 383 KB)

## References

[1] Oishi MM, Robley R, Inada MK, Hiramoto J (2022). Anti-racist approaches to increase access to general and oral health care during a pandemic in the Pacific Islander community. J Public Health Dent..

[2] Abubakar I, Gram L, Lasoye S (2022). Confronting the consequences of racism, xenophobia, and discrimination on health and health-care systems. Lancet..

[3] Williams N, Sulistio MS, Winchester DE, Chen C, Toft LEB (2021). How to Build an Antiracist Cardiovascular Culture, Community, and Profession. J Am Coll Cardiol..

[4] Swartz TH, Palermo AS, Masur SK, Aberg JA (2019). The Science and Value of Diversity: Closing the Gaps in Our Understanding of Inclusion and Diversity. J Infect Dis..

[5] Dimitropoulos G, Bright KS, Li QKW (2022). Equity, diversity and inclusion of pediatric clinician-scientists in Canada: a thematic analysis. CMAJ Open..

[6] Daldrup-Link HE, Esposito G, Bhujwalla ZM (2021). Challenges and Initiatives in Diversity, Equity and Inclusion in Cancer Molecular Imaging. Front Oncol..

[7] Schwartz AL, Alsan M, Morris AA, Halpern SD (2023). Why Diverse Clinical Trial Participation Matters. N Engl J Med..

[8] Tahhan AS, Vaduganathan M, Greene SJ (2018). Enrollment of Older Patients, Women, and Racial and Ethnic Minorities in Contemporary Heart Failure Clinical Trials: A Systematic Review. JAMA Cardiol..

[9] Tahhan AS, Vaduganathan M, Greene SJ (2020). Enrollment of Older Patients, Women, and Racial/Ethnic Minority Groups in Contemporary Acute Coronary Syndrome Clinical Trials: A Systematic Review. JAMA Cardiol..

[10] Muthukumar AV, Morrell W, Bierer BE (2021). Evaluating the frequency of English language requirements in clinical trial eligibility criteria: A systematic analysis using ClinicalTrials.gov. PLoS Med..

[11] Elliott T, Floyd James K, Coleman KJ, Skrine Jeffers K, Nau CL, Choi K (2022). Cross-sectional Comparison of Disparities by Race Using White vs Hispanic as Reference Among Children and Youths With Developmental Disabilities Referred for Speech Therapy. JAMA Netw Open..

[12] Ramírez M, Ford ME, Stewart AL, Teresi JA (2005). Measurement issues in health disparities research. Health Serv Res..

[13] Volpe VV, Dawson DN, Rahal D, Wiley KC, Vesslee S (2019). <h2 _ngcontent-lwb-c132="" class="title" style="box-sizing: border-box; font-family: sans-serif; font-weight: 500; line-height: 1.1; color: rgb(44, 114, 183); margin-top: 20px; margin-bottom: 10px; background-color: rgb(255, 255, 255);">Bringing psychological science to bear on racial health disparities: The promise of centering Black health through a critical race framework. Transl Issues Psychol Sci..

[14] Boyd RW, Lindo EG, Weeks LD, McLemore MR (2020). On Racism: A New Standard For Publishing On Racial Health Inequities. Health Aff Forefront..

[15] Summer Undergraduate Mentored Research Program (SUMR). Leonard Davis Institute of Health Economics,. Accessed March 30, 2023. https://ldi.upenn.edu/education/penn-ldi-training-programs/sumr/.

[16] Addressing equity in research participation incentives. University of Michigan Institute for Research on Women and Gender. Accessed March 3, 2023. https://irwg.umich.edu/news/addressing-equity-research-participation-incentives.

[17] Beyond altruism - exploring payment for research participation. OHRP Exploratory Workshop, HHS. Accessed March 20, 2023. https://www.hhs.gov/sites/default/files/oew-program-book-2022.pdf.

[18] Bierer BE, White SA, Gelinas L, Strauss DH (2021). Fair payment and just benefits to enhance diversity in clinical research. J Clin Transl Sci..

[19] **Cheff R, Roche B.** Considerations for compensating research participants fairly & equitably: A think piece. Wellesley Institute. Accessed July 26, 2023. https://www.wellesleyinstitute.com/wp-content/uploads/2018/07/Fair-compensation-Think-Piece-.pdf.

[20] Shy O (2020). Low-income consumers and payment choice. Res Econ..

[21] Eshun-Wilson I, Akama E, Adhiambo F (2022). Adolescent and young adult preferences for financial incentives to support adherence to antiretroviral therapy in Kenya: a mixed methods study. J Int AIDS Soc..

[22] **Foster K, Greene C, Stavins J.** The 2021 Survey and Diary of Consumer Payment Choice: Summary Results. Federal Reserve Bank of Atlanta. Accessed July 26, 2023. https://www.atlantafed.org/-/media/documents/banking/consumer-payments/survey-diary-consumer-payment-choice/2021/sdcpc_2021_report.pdf.

[23] Gelinas L, Largent EA, Cohen IG, Kornetsky S, Bierer BE, Fernandez Lynch H (2018). A Framework for Ethical Payment to Research Participants. N Engl J Med..

[24] Grady C, Dickert N, Jawetz T, Gensler G, Emanuel E (2005). An analysis of U.S. practices of paying research participants. Contemp Clin Trials..

[25] Grady C (2005). Payment of clinical research subjects. J Clin Invest..

[26] Halpern SD, Karlawish JH, Casarett D, Berlin JA, Asch DA (2004). Empirical assessment of whether moderate payments are undue or unjust inducements for participation in clinical trials. Arch Intern Med..

[27] Halpern SD, Madison KM, Volpp KG (2009). Patients as mercenaries?: the ethics of using financial incentives in the war on unhealthy behaviors. Circ Cardiovasc Qual Outcomes..

[28] Halpern SD, Chowdhury M, Bayes B (2021). Effectiveness and Ethics of Incentives for Research Participation: 2 Randomized Clinical Trials. JAMA Intern Med..

[29] Jennings CG, MacDonald TM, Wei L, Brown MJ, McConnachie L, Mackenzie IS (2015). Does offering an incentive payment improve recruitment to clinical trials and increase the proportion of socially deprived and elderly participants?. Trials..

[30] Largent EA, Grady C, Miller FG, Wertheimer A (2012). Money, coercion, and undue inducement: attitudes about payments to research participants. IRB..

[31] Largent E, Grady C, Miller FG, Wertheimer A (2013). Misconceptions about coercion and undue influence: reflections on the views of IRB members. Bioethics..

[32] Largent EA, Fernandez Lynch H (2017). Paying Research Participants: Regulatory Uncertainty, Conceptual Confusion, and a Path Forward. Yale J Health Policy Law Ethics..

[33] Fernandez Lynch H, Joffe S, Thirumurthy H, Xie D, Largent EA (2019). Association Between Financial Incentives and Participant Deception About Study Eligibility. JAMA Netw Open..

[34] Ngo S, Kim AS, Chiong W (2021). Evidence for the Ethics of Incentivizing Clinical Trial Enrollment?. JAMA Intern Med..

[35] **O'Brien S.** Consumer preferences and the use of cash: Evidence from the diary of consumer payments choice. Federal Reserve Bank of San Francisco. Accessed July 26, 2023. https://www.frbsf.org/cash/publications/fed-notes/2014/july/consumer-preferences-cash-use/.

[36] Noordraven EL, Schermer MHN, Blanken P, Mulder CL, Wierdsma AI (2017). Ethical acceptability of offering financial incentives for taking antipsychotic depot medication: patients' and clinicians' perspectives after a 12-month randomized controlled trial. BMC Psychiatry..

[37] Resnik DB (2015). Bioethical Issues in Providing Financial Incentives to Research Participants. Medicoleg Bioeth..

[38] Slomka J, McCurdy S, Ratliff EA, Timpson S, Williams ML (2007). Perceptions of financial payment for research participation among African-American drug users in HIV studies. J Gen Intern Med..

[39] Walter JK, Burke JF, Davis MM (2013). Research participation by low-income and racial/ethnic minority groups: how payment may change the balance. Clin Transl Sci..

[40] Williams EP, Walter JK (2015). When Does the Amount We Pay Research Participants Become "Undue Influence"?. AMA J Ethics..

[41] Zutlevics TL (2016). Could providing financial incentives to research participants be ultimately self-defeating?. Res Ethics..

[42] United States Census Bureau - Detailed languages spoken at home and ability to speak English for the population 5 years and over: 2022. Accessed November 6, 2023. https://data.census.gov/table/ACSDP1Y2022.DP02.

[43] 2015 Language Map App. LEP.gov. Accessed July 27, 2023. https://www.lep.gov/maps/lma2015/Final.

[44] Alhalel J, Francone N, Post S, O'Brian CA, Simon MA (2022). How Should Representation of Subjects With LEP Become More Equitable in Clinical Trials?. AMA J Ethics..

[45] Commonly Asked Questions. LEP.gov. Accessed July 27, 2023. https://www.lep.gov/commonly-asked-questions.

[46] Enrolling Participants with Limited English Proficiency (LEP) & Short Consent Form Translations. University of Pennsylvania Institutional Review Board. Accessed July 27, 2023. https://irb.upenn.edu/homepage/biomedical-homepage/guidance/recruitment-andconsent/.

[47] Resnik DB, Jones CW (2006). Research subjects with limited English proficiency: ethical and legal issues. Account Res..

[48] **Dietrich S, Hernandez E.** Nearly 68 Million People Spoke a Language Other Than English at Home in 2019. United States Census Bureau. Accessed July 27, 2023. https://www.census.gov/library/stories/2022/12/languages-we-speak-in-united-states.html.

[49] The Terminology of Health Care Interpreting: A Glossary of Terms. The National Council on Interpreting in Health Care (NCIHC). Accessed July 27, 2023. https://www.ncihc.org/assets/documents/NCIHC%20Terms%20Final080408.pdf.

[50] What's in a Word? A Guide to Understanding Interpreting and Translation in Health Care. National Council on Interpreting in Health Care (NCIHC), American Translators Association (ATA), National Health Law Program (NHeLP). Accessed July 27, 2023. https://www.ncihc.org/assets/documents/publications/Whats_in_a_Word_Guide.pdf.

[51] Programs. Migration Policy Institute (MPI). Accessed July 27, 2023. https://www.migrationpolicy.org/programs/language%C2%.

[52] March 2023 Medicaid & CHIP Enrollment Data Highlights. Medicaid.gov. Accessed July 27, 2023. https://www.medicaid.gov/medicaid/program-information/medicaid-and-chip-enrollment-data/report-highlights/index.html.

[53] Brodeur M, Herrick J, Guardioloa J, Richman P (2017). Exclusion of Non-English Speakers in Published Emergency Medicine Research - A Comparison of 2004 and 2014. Acta Inform Med..

[54] Flores G (2005). The impact of medical interpreter services on the quality of health care: a systematic review. Med Care Res Rev..

[55] Sanossian N, Rosenberg L, Liebeskind DS (2017). A Dedicated Spanish Language Line Increases Enrollment of Hispanics Into Prehospital Clinical Research. Stroke..

[56] Guide to Developing a Language Access Plan. Centers for Medicare & Medicaid Services (CMS). Accessed July 27, 2023. https://www.cms.gov/About-CMS/Agency-Information/OMH/Downloads/Language- Access-Plan-508.pdf.

[57] Types of Interpreters: Benefits & Limitations. Stanford Medicine Ethnogeriatrics. Accessed July 27, 2023. https://geriatrics.stanford.edu/culturemed/overview/assessment/interpreter_types.html.

[58] Giordano S (2007). Overview of the Advantages and Disadvantages of Professional and Child Interpreters for Limited English Proficiency Patients in General Health Care Situations. J Radiol Nurs..

[59] Guidance to Federal Financial Assistance Recipients Regarding Title VI Prohibition Against National Origin Discrimination Affecting Limited English Proficient Persons. Department of Justice. Accessed July 27, 2023. https://www.federalregister.gov/documents/2002/06/18/02-15207/guidance-to-federal-financialassistance-.

[60] **Godfrey L.** Lost in Translation. Digital.gov. Accessed July 27, 2023. https://digital.gov/2012/10/01/automated-translation-good-solution-or-not/.

[61] Health Literacy Online. Office of Disease Prevention and Health Promotion. Accessed July 26, 2023. https://health.gov/healthliteracyonline/.

[62] Health Literacy Guidance. University of Pennsylvania Institutional Review Board. Accessed July 26, 2023. https://irb.upenn.edu/wp-content/uploads/2023/02/Health-Literacy-Guidance.pdf.

[63] plainlanguage.gov. Plain Language Action and Information Network (PLAIN); U.S. General Services Administration. Accessed March 28, 2023. https://www.plainlanguage.gov/.

[64] The Effectiveness of Plain Language Proven by Data. BVA/Labrador Language Services. Accessed July 26, 2023. http://img.mailinblue.com/2103085-381/attachments/LABRADORBVAPLAINLANGUAGESTUDY2020.pdf.

[65] Areas of Responsibility, Competencies and Sub-competencies for Health Education Specialist Practice Analysis II 2020 (HESPA II 2020). National Commission for Health Education Credentialing (NCHEC). Accessed July 26, 2023. https://t8537154.p.clickup-attachments.com/t8537154/6df1b186-c921-4b7c-988c-6487fbb70f98/hespa_competencies_and_sub-competencies_2020.pdf?view=open.

[66] Readability Calculator. WordCalc. Accessed July 26, 2023. https://www.wordcalc.com/readability/.

[67] The Flesch Grade Level Readability Formula. Accessed July 26, 2023. https://readabilityformulas.com/flesch-grade-level-readability-formula.php.

[68] The SMOG Readability Formula, a Simple Measure of Gobbledygook. Accessed July 26, 2023. https://readabilityformulas.com/smog-readability-formula.php.

[69] **Shoemaker SJ, Wolf MS, Brach C.** Patient Education Materials Assessment Tool for Printable Materials (PEMAT-P). Agency for Healthcare Research and Quality. https://www.ahrq.gov/sites/default/files/publications/files/pemat-p.pdf. Accessed 26 Jul 2023.

[70] GLAAD Media Reference Guide 11th Edition. GLAAD. Accessed July 31, 2023. https://glaad.org/reference/.

[71] Bias-Free Language. American Psychological Association. Accessed April 7, 2023. https://apastyle.apa.org/style-grammar-guidelines/bias-free-language.

[72] Advancing Health Equity: A Guide to Language, Narrative and Concepts. American Medical Association and Association of American Medical Colleges. Accessed April 7, 2023. https://www.ama-assn.org/system/files/ama-aamc-equity-guide.pdf.

[73] Race, Ethnicity, and Language Data: Standardization for Health Care Quality Improvement. 2009. https://www.ncbi.nlm.nih.gov/books/NBK219756. Accessed 7 Apr 2023. 25032349

[74] AMA Manual of Style 11th Edition: A Guide for Authors and Editors. 11th ed. Oxford Academic and JAMA Network; 2020. https://academic.oup.com/amamanualofstyle/book/27941. Accessed 31 Jul 2023.

[75] Amutah C, Greenidge K, Mante A (2021). Misrepresenting Race - The Role of Medical Schools in Propagating Physician Bias. N Engl J Med..

[76] Equity, Diversity, and Inclusion: Inclusive Language Guidelines. American Psychological Association. Accessed July 31, 2023. https://www.apa.org/about/apa/equity-diversity-inclusion/language-guidelines.pdf.

[77] Suen LW, Lunn MR, Katuzny K (2020). What Sexual and Gender Minority People Want Researchers to Know About Sexual Orientation and Gender Identity Questions: A Qualitative Study. Arch Sex Behav..

[78] Dickinson JK, Guzman SJ, Maryniuk MD (2017). The Use of Language in Diabetes Care and Education. Diabetes Care..

[79] Guidelines for Writing About People With Disabilities. Americans with Disabilities Act (ADA) National Network. Accessed July 31, 2023. https://adata.org/factsheet/ADANN-writing.

[80] Adamson AS, Lipoff JB (2021). Reconsidering Named Honorifics in Medicine-the Troubling Legacy of Dermatologist Albert Kligman. JAMA Dermatol..

[81] **Jones-Alston AD.** My family needs more from Penn than an apology for unethical medical experiments The Philadelphia Inquirer. https://www.inquirer.com/opinion/commentary/penn-medicine-apology-albert-kligman-experiments-20210826.html#loaded. Accessed 27 Mar 2023.

