# Supplementary material for: Operationalizing Equity, Inclusion, and Access in Research Practice at a Large Academic Institution
Source: J Gen Intern Med. 2024 Feb 1;39(6):1037–47. doi: 10.1007/s11606-023-08539-z (PMC11074076; doi:10.1007/s11606-023-08539-z)
Supplement: Supplementary file 1 — Supplementary file1 (DOCX 383 KB) [file 11606_2023_8539_MOESM1_ESM.docx]

**Operationalizing equity, inclusion, and access**

**in research practice at a large academic institution**

**SUPPLEMENTARY MATERIALS**

**Table of contents**

| **Section** | **Page** |
| --- | --- |
| Supplementary Figure | 2 |
| Supplementary Tables | 3 |

**SUPPLEMENTARY FIGURE**

**eFigure 1. The Joint Research Practices Working Group multi-pronged approach using mutual learning**

**
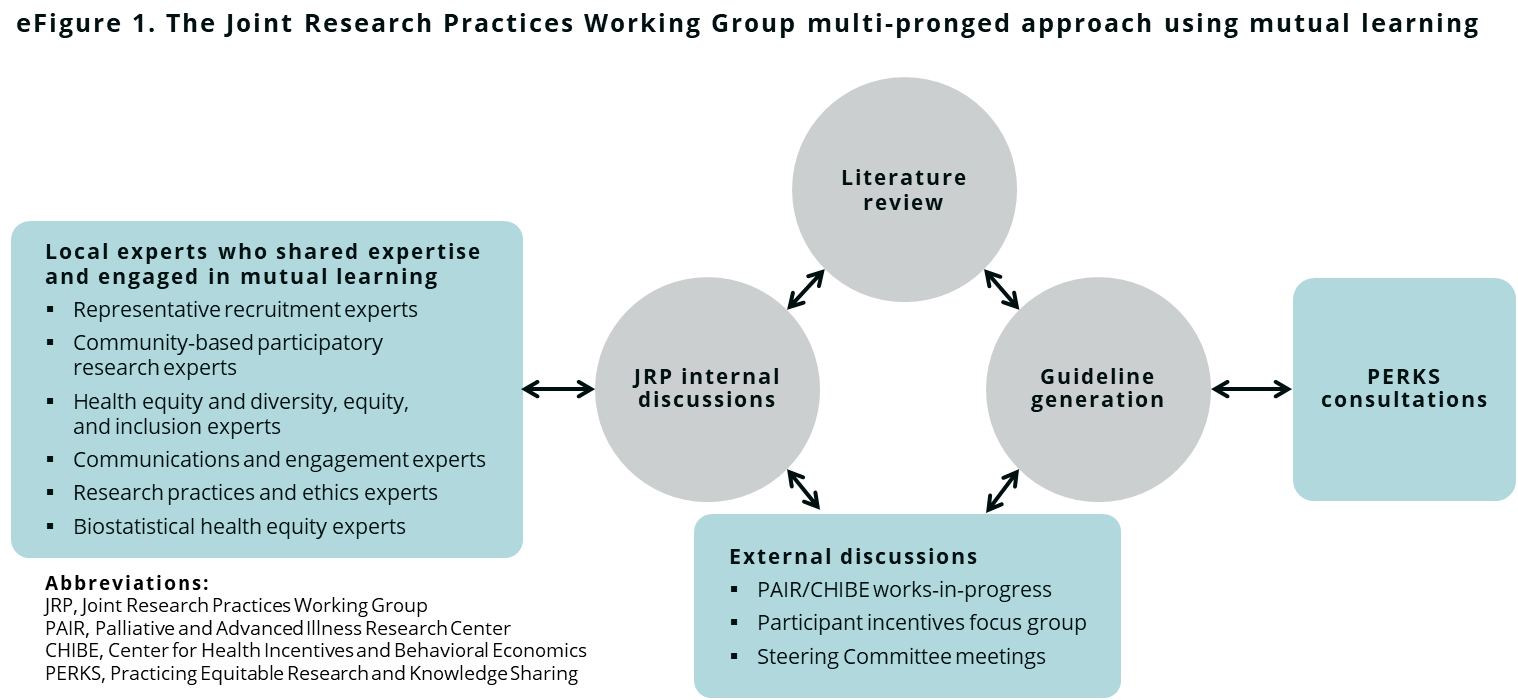
**

**SUPPLEMENTARY TABLES**

**eTable 1. Multi-pronged approaches by topic area**

**
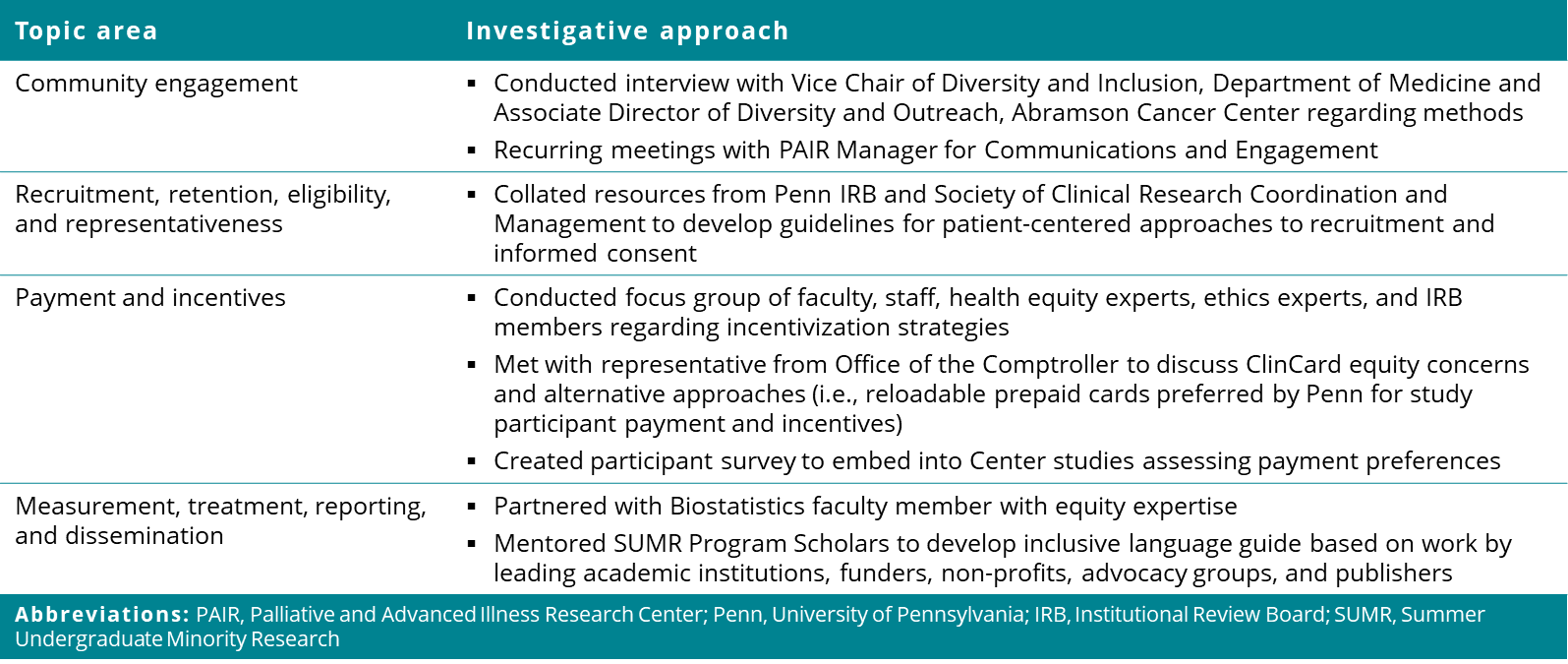
**

**eTable 2. Conference workshop 2022: Holmesburg Prison Experiments breakout session discussion questions**

**
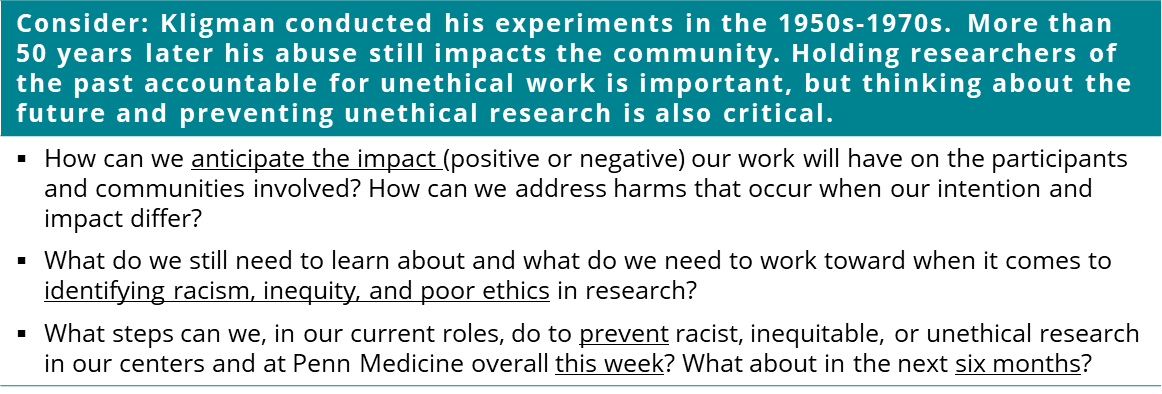
**

**eTable 3. Conference workshop 2022: Breakout session on key topic areas**

**
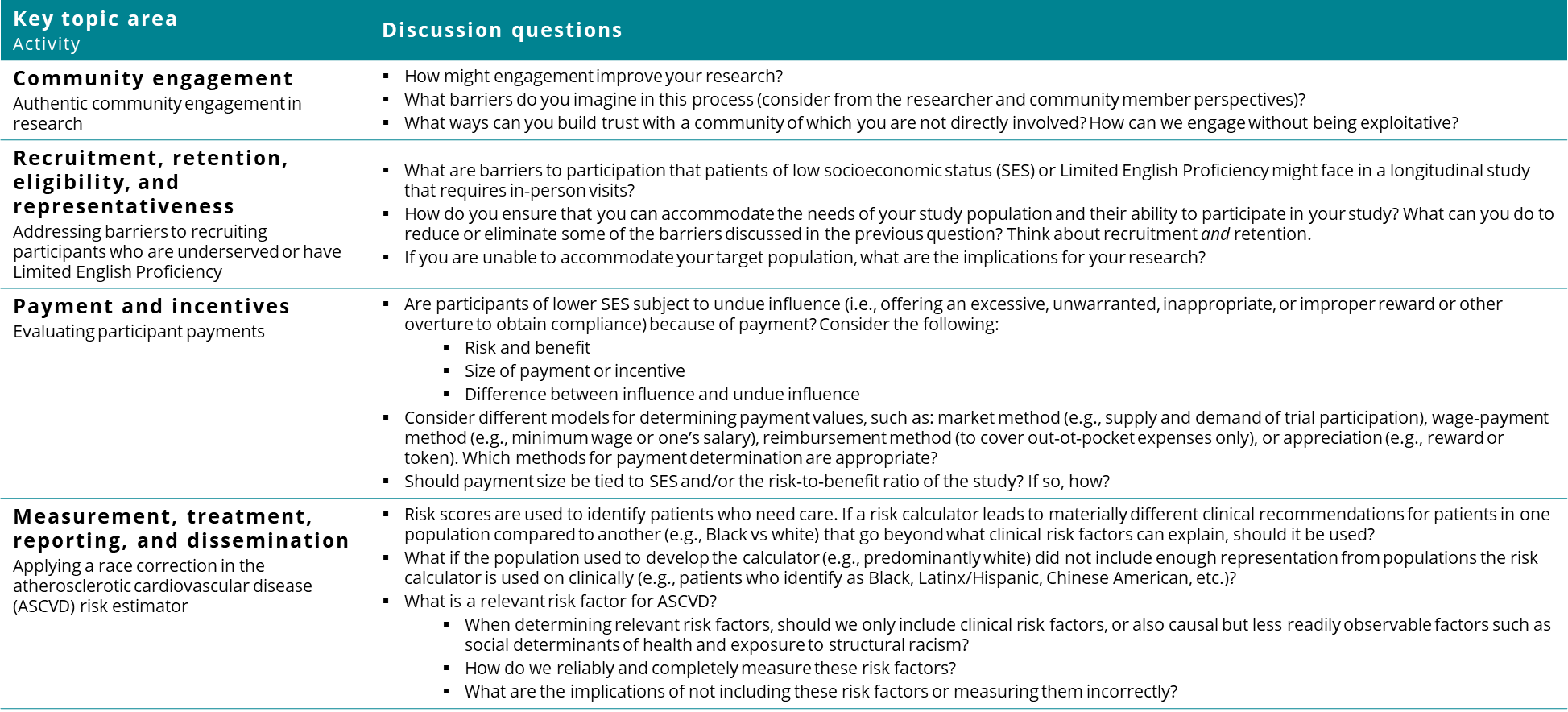
**

**eTable 4. Conference workshop 2023: Breakout session topic areas and example commitments**

**
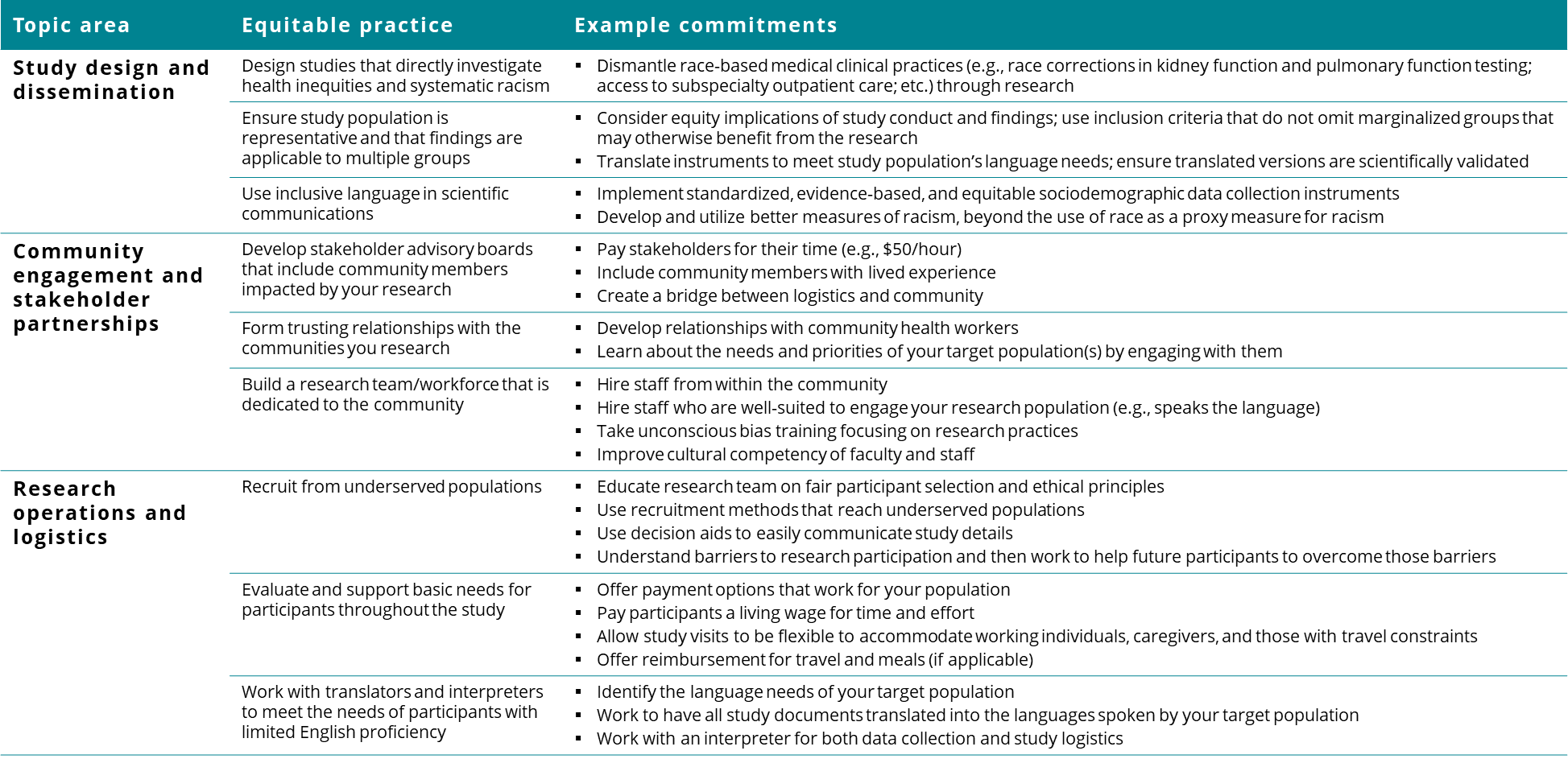
**

**eTable 5. Conference workshop 2023: Breakout session prompts to create personal commitments**

**
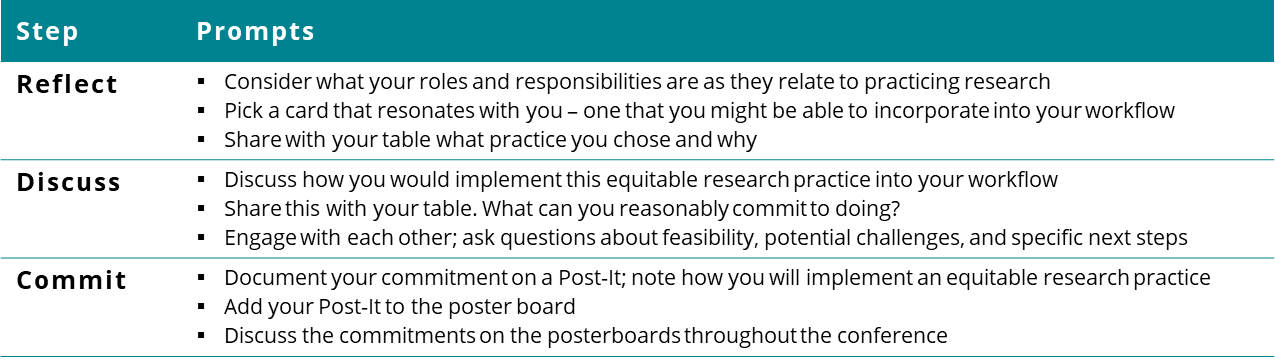
**
